# Iron-Fueled Life in the Continental Subsurface: Deep Mine Microbial Observatory, South Dakota, USA

**DOI:** 10.1128/AEM.00832-21

**Published:** 2021-09-28

**Authors:** C. P. Casar, L. M. Momper, B. R. Kruger, M. R. Osburn

**Affiliations:** a Department of Earth and Planetary Sciences, Northwestern University, Evanston, Illinois, USA; b Earth and Environmental Sciences Practice, Exponent, Inc., Pasadena, California, USA; c Division of Hydrologic Sciences, Desert Research Institute, Las Vegas, Nevada, USA; University of Michigan-Ann Arbor

**Keywords:** DeMMO, FeGenie, continental subsurface, deep subsurface, iron cycling

## Abstract

Iron-bearing minerals are key components of the Earth’s crust and potentially critical energy sources for subsurface microbial life. The Deep Mine Microbial Observatory (DeMMO) is situated in a range of iron-rich lithologies, and fracture fluids here reach concentrations as high as 8.84 mg/liter. Iron cycling is likely an important process, given the high concentrations of iron in fracture fluids and detection of putative iron-cycling taxa via marker gene surveys. However, a previous metagenomic survey detected no iron cycling potential at two DeMMO localities. Here, we revisited the potential for iron cycling at DeMMO using a new metagenomic data set including all DeMMO sites and FeGenie, a new annotation pipeline that is optimized for the detection of iron cycling genes. We annotated functional genes from whole metagenomic assemblies and metagenome-assembled genomes and characterized putative iron cycling pathways and taxa in the context of local geochemical conditions and available metabolic energy estimated from thermodynamic models. We reannotated previous metagenomic data, revealing iron cycling potential that was previously missed. Across both metagenomic data sets, we found that not only is there genetic potential for iron cycling at DeMMO, but also, iron is likely an important source of energy across the system. In response to the dramatic differences we observed between annotation approaches, we recommend the use of optimized pipelines where the detection of iron cycling genes is a major goal.

**IMPORTANCE** We investigated iron cycling potential among microbial communities inhabiting iron-rich fracture fluids to a depth of 1.5 km in the continental crust. A previous study found no iron cycling potential in the communities despite the iron-rich nature of the system. A new tool for detecting iron cycling genes was recently published, which we used on a new data set. We combined this with a number of other approaches to get a holistic view of metabolic strategies across the communities, revealing iron cycling to be an important process here. In addition, we used the tool on the data from the previous study, revealing previously missed iron cycling potential. Iron is common in continental crust; thus, our findings are likely not unique to our study site. Our new view of important metabolic strategies underscores the importance of choosing optimized tools for detecting the potential for metabolisms like iron cycling that may otherwise be missed.

## INTRODUCTION

Iron is an essential element sustaining microbial life in the deep continental subsurface. Iron is the fourth most abundant element in the continental crust, averaging 4.32% of bulk crustal composition (1), and aqueous species can be abundant in deep crustal fluids (2). Iron is also an essential nutrient for all life, forming key components of metalloproteins critical to electron transfer in many biological processes and provides a source of metabolic energy for iron-cycling bacteria and archaea (2–5). Despite its ubiquity and importance for the deep continental biosphere, the role of iron as an ecological driver of microbial diversity and metabolic function in deep continental subsurface systems is not well characterized.

This lack of characterization is in part due to the challenges surrounding performing metagenomic surveys in deep continental settings in general as well as iron specific challenges. Accessing deep crustal fluids to kilometer depths in itself can be a technically complex endeavor, and prevalent low cell densities often result in prohibitively low biomass recovery for DNA sequencing. Where metagenomic data sets have been produced, annotating these data requires the use of automated pipelines and careful consideration of databases for gene querying. In the case of detecting iron-specific genes, this can be problematic, as popular gene annotation pipelines, including RAST, GhostKOALA, and MAPLE, are known to overlook genes involved in iron cycling due to lack of gene representation in the queried database (6). This issue can be remedied through the use of new annotation pipelines that are optimized for detecting iron cycling genes of interest.

The Deep Mine Microbial Observatory (DeMMO) is a network of boreholes situated within an iron-rich continental subsurface system intersecting iron-rich metabasalt, siderite- to grunerite-based iron formation, and pyrite-rich schist units. DeMMO boreholes tap fracture fluids to a depth of 1.5 km that have been monitored for geochemistry and microbial community composition since 2014. The sites are geochemically stable and reflect the host rock chemistry and water flow paths. DeMMO sites are locally rich in dissolved iron, reaching values as high as 8.84 mg/liter (2). Although iron-fueled metabolisms are energetically favorable at DeMMO (2, 7), a previous metagenomic survey of two boreholes on the deepest level found no metabolic potential for iron reduction or oxidation by the planktonic communities (8). High concentrations of iron in the fracture fluids and abundant putative iron cycling taxa detected in previous 16S rRNA gene surveys suggest that iron cycling is likely an important process sustaining microbial life here, contradicting previous metagenomic results (2, 9, 10). A recently developed annotation pipeline, FeGenie, incorporates a highly curated, comprehensive database of profile hidden Markov models (HMMs) specific to iron metabolisms and has the potential to elucidate previously undetected iron cycling capacity within the planktonic communities at DeMMO (6).

Here, we revisited the potential for iron-fueled life at DeMMO using a new metagenomic survey of microbes filtered from fracture fluids from all six DeMMO sites which span a range of iron concentrations and depths. We characterized the thermodynamic and functional potential for dissimilatory iron oxidation and reduction and explored the specific taxa involved in iron cycling at each site. Using these data, we explored potential metabolic pathways for iron cycling, addressed whether thermodynamic predictions align with functional potential, and determined which members of the community are capable of iron cycling in this deep continental setting.

## RESULTS

### Metagenomic functional profiles.

**FIG 1:**
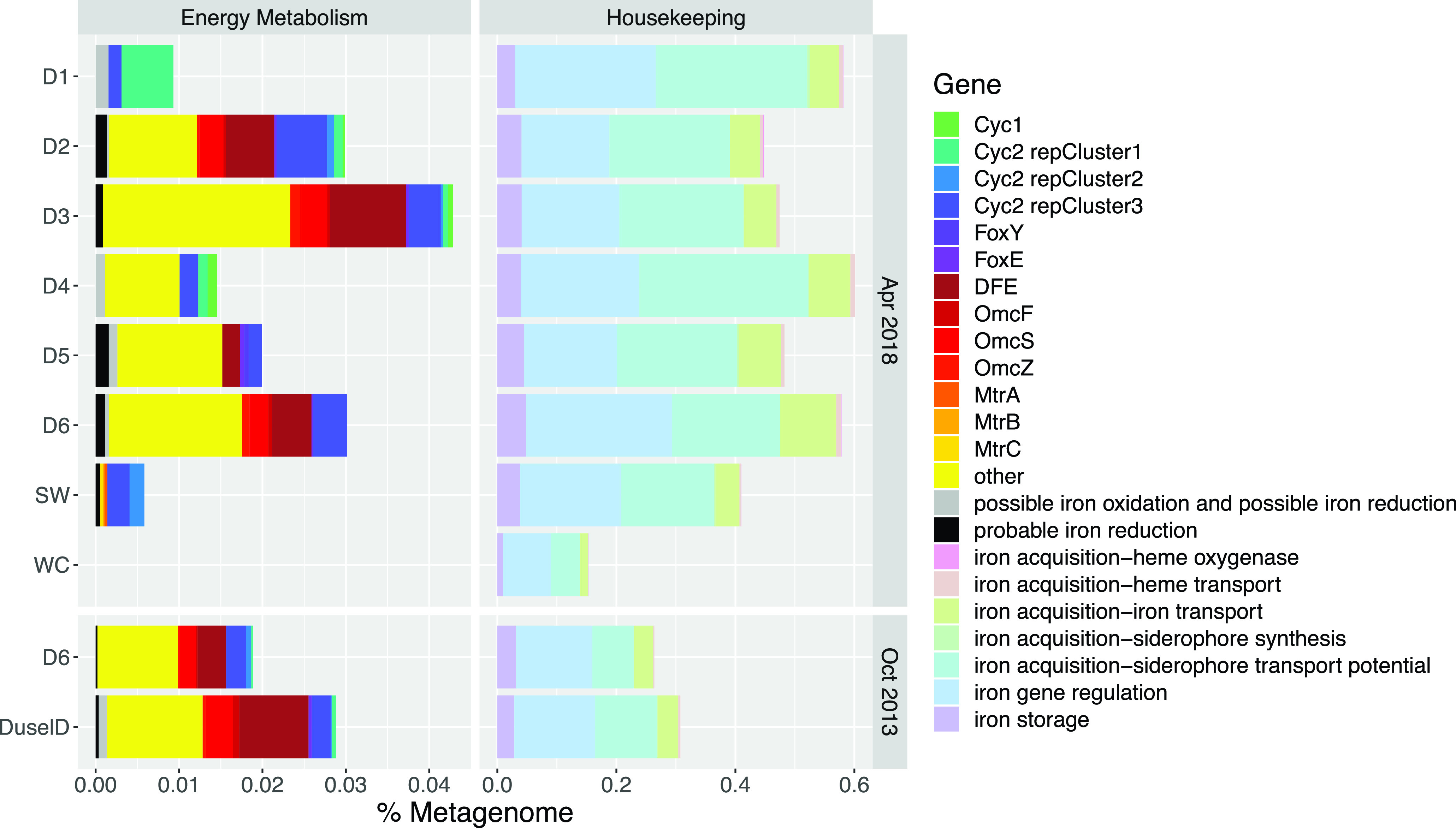
Relative abundances of iron cycling genes annotated via FeGenie. Saturated cool colors denote genes for iron oxidation; warm colors show those for iron reduction. “Other” represents hypothetical proteins attributed to porins and cytochromes for iron reduction. Pastels denote housekeeping genes related to iron acquisition, storage, and regulation. Note that genes corresponding to “iron acquisition–siderophore synthesis” are not visible here due to their low relative abundance.

We annotated metagenomic assemblies with FeGenie to explore the potential for iron cycling among DeMMO and control communities. Across DeMMO and control communities, genes for energy metabolisms with iron vary with iron concentrations. Overall, the highest relative abundances of genes for energy metabolisms with iron occur among D2, D3, and D6 communities (Fig. 1). The lowest relative abundance of these genes occurs in the service water (SW) control community, and none were detected via FeGenie in the Whitewood Creek (WC) surface control community. Genes corresponding to iron oxidation were detected among DeMMO and SW control communities and are dominantly Cyc2 genes encoding fused cytochrome-porins. D1 genes are dominantly Cyc2 representative cluster 1, whereas iron oxidation genes in the other communities are dominantly Cyc2 representative cluster 3. Iron reduction genes were detected among D2 to D6 and SW control communities. D2, D3, and D6 communities have similar relative abundances of OmcF, OmcS, and OmcZ genes encoding outer membrane *c*-type cytochromes, whereas these genes were not detected in D4 and D5 communities. DFE genes encoding encode multiheme cytochromes were detected in similar relative abundances among D2, D3, D5, and D6 communities. Iron reduction genes categorized as “other” include those encoding hypothetical proteins attributed to porins and cytochromes and were detected in similar relative abundances among D2 to D6 communities.

Although the proportions of housekeeping genes relative to each other are generally constant across communities, the overall relative abundances of these genes are low in surface control relative to subsurface DeMMO and SW communities. High relative abundances of genes related to siderophore transport potential, including those encoding TonB, ExbB, and ExbD, iron regulation genes, including that for PchR, and genes related to iron regulation, including those within the Fur family, occur in DeMMO communities (see Table S3 in the supplemental material).

**FIG 2:**
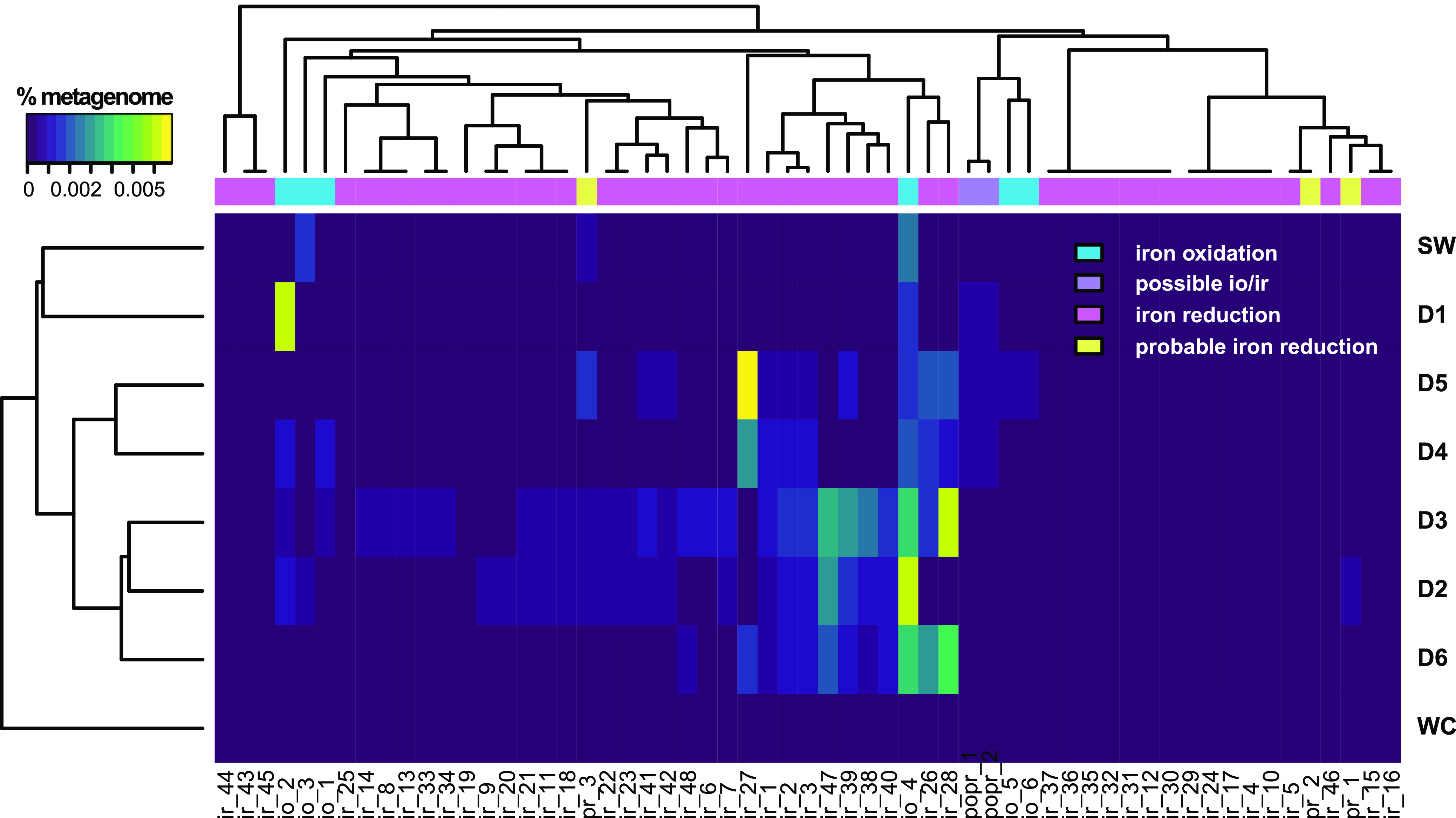
Heat map of genes corresponding to energy metabolisms with iron annotated via FeGenie with hierarchical clustering. Gene names corresponding to *x* axis gene IDs are provided in Table S4.

Hierarchical clustering of DeMMO sites and control communities annotated via FeGenie reveals three distinct clades comprising sites D2, D3, and D6, sites D4 and D5, and site D1 and service water (Fig. 2). The Whitewood Creek surface control community forms an outgroup due to the lack of detected iron oxidation or reduction genes. Relative abundances of genes for iron oxidation and reduction differ significantly among these three groups. The D1 and service water communities are distinguished by their lack of iron reduction genes. D4 and D5 communities possess genes for both iron reduction and oxidation; however, these genes are present in lower relative abundances and are composed of a different assemblage of genes than those found in sites D2, D3 and D6. Indeed, the potential for iron cycling is greatest and most diverse at sites D2, D3, and D6.

**FIG 3:**
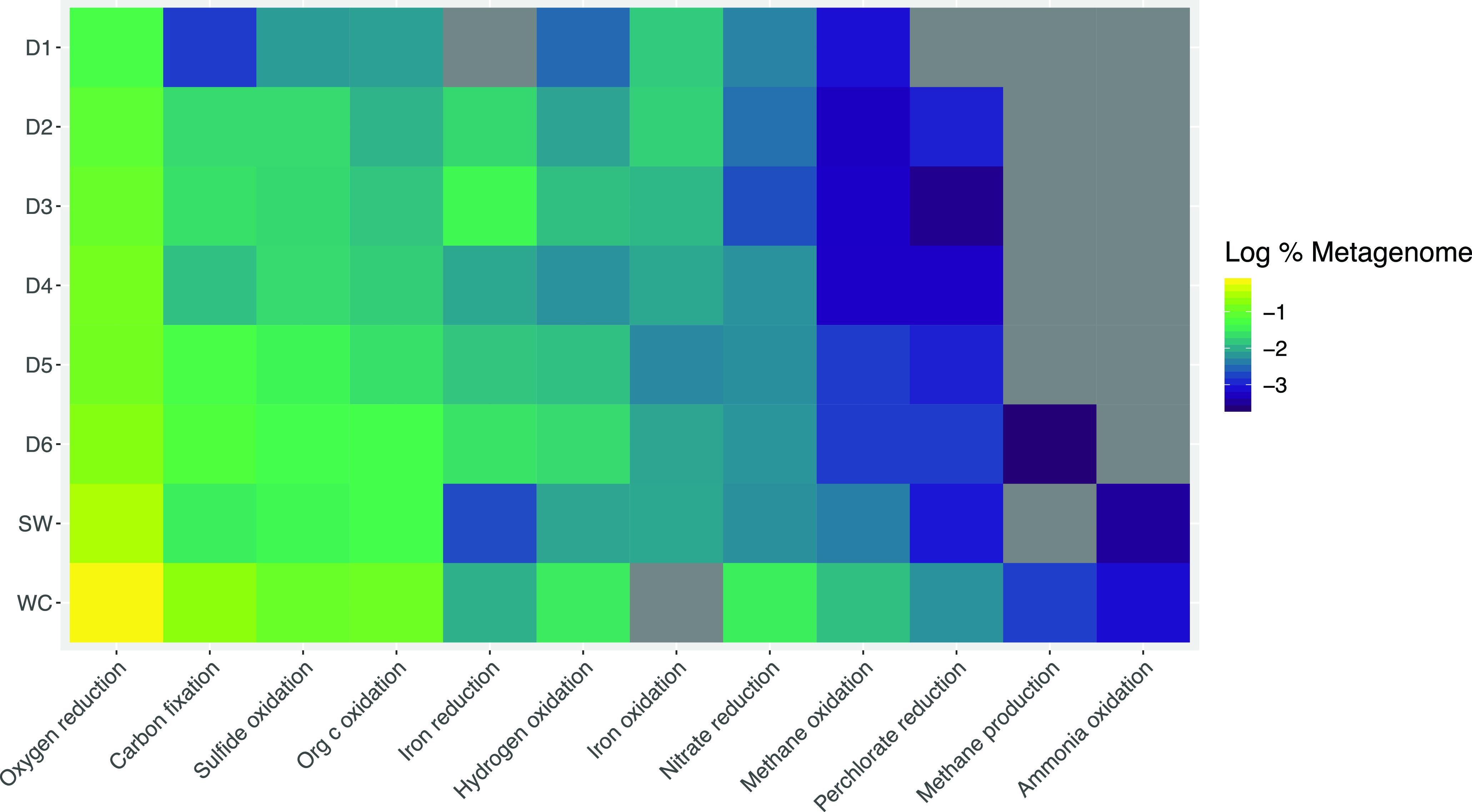
Energy metabolism pathways in DeMMO communities from combined FeGenie and METABOLIC annotations. Scale is log of relative abundances of genes per metagenome; gray indicates that the pathway was not detected via FeGenie or METABOLIC pipelines. “Oxygen reduction” includes “Oxidative phosphorylation” and “Oxygen Metabolism (Oxidative phosphorylation Complex IV)” categories from METABOLIC. “Hydrogen oxidation” includes the “Hydrogenases” category from METABOLIC. “Iron reduction” includes the “iron reduction” category from FeGenie and “Metal reduction” category from METABOLIC. “Iron oxidation” includes the “iron oxidation” category from FeGenie.

We annotated metagenome data via METABOLIC to probe for metabolic pathways that can be coupled to energy metabolisms with iron, including those corresponding to carbon fixation and energy metabolisms with ammonia, hydrogen, nitrate, oxygen, methane, organic carbon, or perchlorate. Communities at all sites possess genes for nitrate reduction, hydrogen oxidation, carbon fixation, oxygen reduction, and organic carbon oxidation, suggesting abundant options for half reactions to pair to iron cycling metabolisms. The highest abundances of these genes occur in the Whitewood Creek surface control community (Fig. 3; Fig. S2), although the lack of genes for iron reduction or oxidation detected via FeGenie here suggests that this metabolic potential is not directly tied to iron cycling. That said, “metal reduction” potential corresponding to *mtrB* and *mtrC* genes detected via METABOLIC represents 0.01% of the WC metagenome. Within DeMMO communities, the highest abundances of genes for carbon fixation and oxidation and nitrate reduction occur in the D6 community. Genes for nitrate reduction and methane oxidation are most abundant among the deeper sites D4 to D6 and in the service water control communities. Genes for ammonia oxidation are in low abundance or were not detected across all sites. While further support for putative metabolic pathways with iron can be inferred from pathways identified in metagenome-assembled genomes (MAGs), we did not attempt this given the wide range of MAG completeness in this data set (Table S5).

### Taxonomic functional potential.

**FIG 4:**
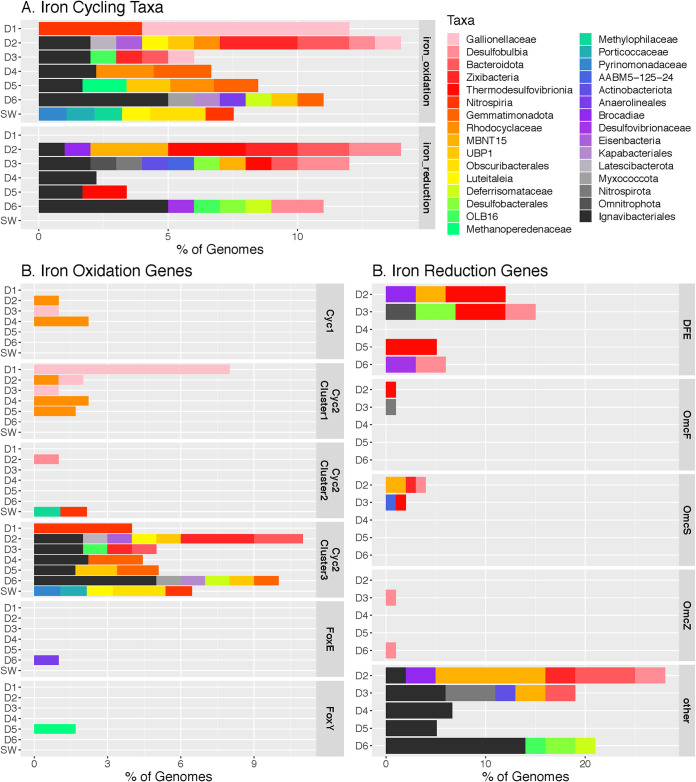
Relative abundances of iron cycling taxa annotated via FeGenie. Only the FeGentie categories “iron oxidation” and “iron reduction” are shown. The “Other” gene category represents hypothetical proteins attributed to porins and cytochromes for iron reduction.

We annotated MAGs to determine which members of the DeMMO and control communities possess iron cycling genes. MAG annotations from FeGenie reported here reflect presence-only data, as MAG completeness varies widely (14 to 100%) (Table S5). Thus, the absence of a gene indicates only that the gene was not detected and does not necessarily mean that the gene is not present in the organism. Broadly, taxa that possess iron cycling genes across D1 to D6 communities are dominantly members of the phyla *Bacteroidota*, *Gemmatimonadota*, *Deferrisomatota*, *Desulfobacterota*, and *Nitrospirota*, class *Gammaproteobacteria*, and several candidate divisions, including the “*Candidatus* Zixibacteria,” *Latescibacterota*, “*Candidatus* Eisenbacteria,” MBNT15, OLB16, and UBP1 (Fig. 4). Taxa that possess iron cycling genes within the service water control community are distinct from those found in D1 to D6 communities and include members of the families *Pyrinomonadaceae*, *Porticoccaceae*, *Methylophilaceae*, and *Obscuribacteraceae*.

Significant taxa that possess genes for iron oxidation include members of the family *Gallionellaceae* at sites D1, D2, and D3 and *Rhodocyclaceae* at sites D2, D4, and D5, both of which possess Cyc2 representative cluster 1 genes encoding fused cytochrome porins. Similarly, notable taxa with genes for iron reduction include members of the class *Thermodesulfovibrionia* at sites D2, D3, and D5 which possess DFE encoding multiheme cytochromes, OmcS and OmcZ genes encoding outer membrane *c*-type cytochromes, and members of the family *Desulfobulbaceae* at sites D3 and D6 which possess DFE and OmcZ genes. Members of the order *Ignavibacteriales* and candidate phylum “*Candidatus* Zixibacteria” possess genes for both iron oxidation and reduction across sites D2 to D6. Among housekeeping genes related to iron acquisition, storage, and regulation detected among the 515 MAGs in this study, 49.5% possess genes in all three categories, 19.8% have genes for iron acquisition and regulation, and 15.7% have genes for only iron regulation. There is no taxonomic or genome completeness trend at the category level among housekeeping genes.

### Metabolic modeling.

We modeled energy metabolisms with iron to determine the energy availability of each reaction under *in situ* conditions at DeMMO. Geochemical data used for thermodynamic models are reported in Fig. 1. Fracture fluids at sites D1 to D3 and D6 are visibly iron rich, with iron floc concentrating on our filters (Fig. S3). No measurable ferrous iron was detected in D4 or D5 fluids via field spectrophotometry, although 0.03 mg/liter total iron was detected in D4 fluids via ion chromatography. In contrast, D4 and D5 fluids are relatively sulfide rich, showing sulfide concentrations 1 to 2 orders of magnitude greater than those of D1 to D3 and D6.

**FIG 5:**
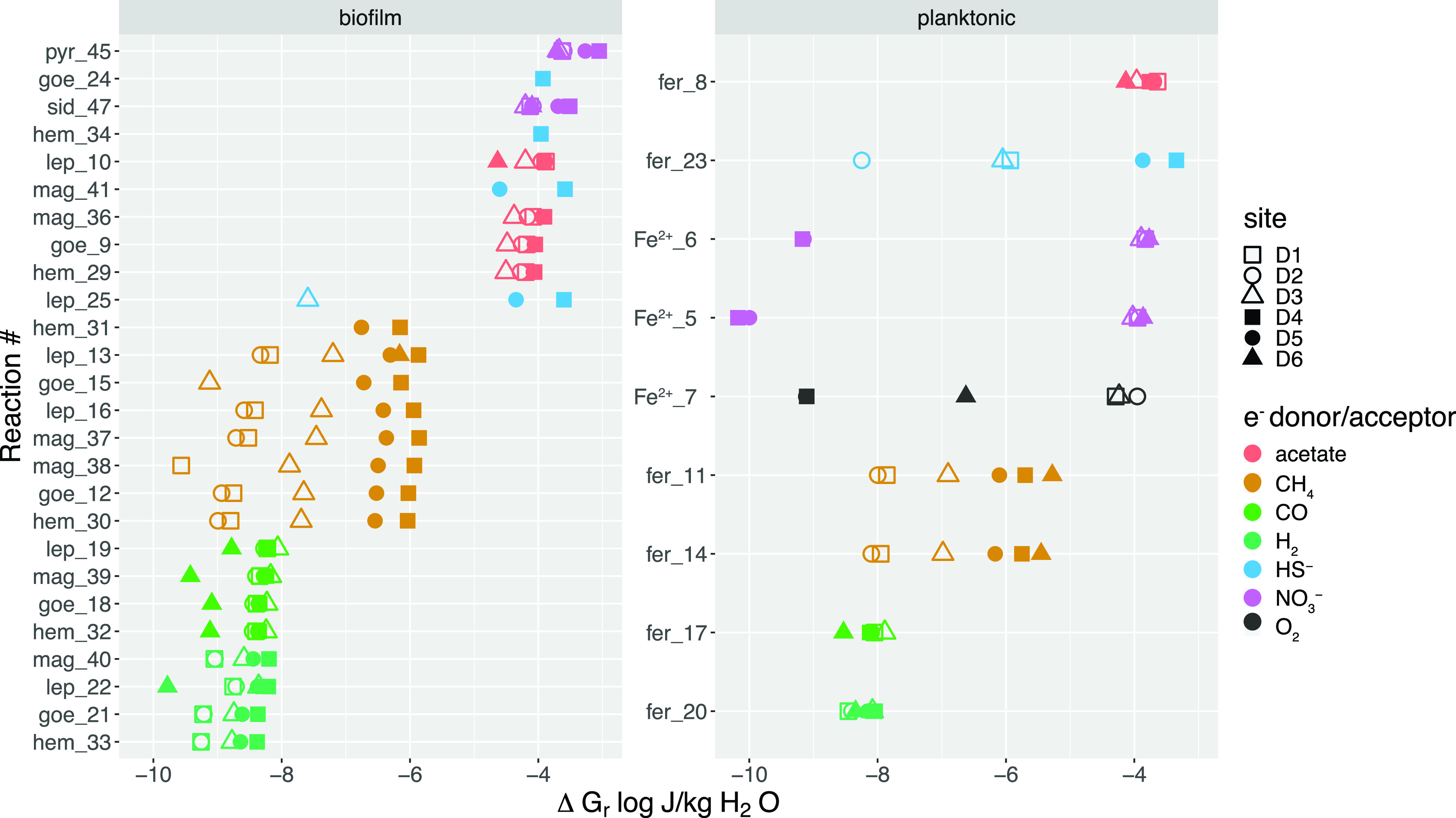
Energy density of metabolic reactions with iron modeled using *in situ* geochemistry is scaled to limiting reactant availability. Reactions that do not appear on this figure are endergonic. Labels on the *y* axis denote the iron species and reaction number, where “pyr,” “sid,” “lep,” “goe,” “hem,” and “fer” correspond to pyrite, siderite, lepidocrocite, goethite, hematite, and ferrihydrite, respectively. Reaction numbers correspond to reactions in Table 2.

Reactions with aqueous Fe^2+^ or ferrihydrite are considered potential metabolisms for members of the planktonic communities given the abundance of ferrous iron and amorphous iron oxides in fracture fluids. All other reactions with more crystalline forms of iron, i.e., goethite, hematite, magnetite, etc., are considered potential metabolisms for members of the biofilm communities on rock surfaces. Reactions with iron minerals are scaled to the availability of the aqueous reactant, and thus, the reported energy densities should be considered upper limits. Several reactions modeled are endergonic, including aqueous Fe^2+^ or pyrite oxidation with bicarbonate, siderite oxidation with sulfate or carbon monoxide, magnetite reduction with ammonium, and hematite reduction with sulfide (Fig. 5).

Among the reactions relevant to planktonic communities, the most metabolic energy is available from aqueous Fe^2+^ oxidation with oxygen at sites D1 to D3 and with nitrate at sites D1 to D3 and D6. Ferrihydrite reduction with acetate has the highest energy yield across all sites and with methane has a moderate energetic yield at the deeper sites D4 to D6. Ferrihydrite reduction with carbon monoxide or hydrogen yields little energy. Aqueous Fe^2+^ oxidation with perchlorate is extremely exergonic per mole of e^−^; however, resultant energy densities are extremely low (Table S6).

Among the reactions relevant to biofilm communities, the most metabolic energy is available from pyrite and siderite oxidation with nitrate and acetate oxidation with lepidocrocite, magnetite, goethite, or hematite across all sites. Hematite and goethite reduction with sulfide are highly energy-yielding metabolisms at D4, and magnetite and lepidocrocite reduction with sulfide are highly energy-yielding metabolisms at sites D4 and D5. Methane oxidation with lepidocrocite, magnetite, goethite, and hematite are all moderately energy yielding metabolisms at sites D4 and D5. Reactions with carbon monoxide or hydrogen as electron donor are low energy-yielding metabolisms.

## DISCUSSION

### Exploring iron cycling potential at DeMMO.

Our previous metabolic models and 16S rRNA gene amplicon surveys indicated that iron cycling is likely an important process sustaining microbial life at DeMMO, despite findings from a previous metagenomic survey. Here, we revisited the potential for iron-fueled life in DeMMO fracture fluid communities across a range of iron concentrations and depths. We modeled energy metabolisms with iron and annotated a new metagenomic data from all six DeMMO sites with FeGenie, a new annotation pipeline optimized for the detection of iron cycling genes. Here, we discuss potential metabolic pathways for iron cycling in the context of thermodynamic predictions and putative iron cycling taxa in this deep continental setting.

Ferrous iron concentrations locally exceed several milligrams per liter at DeMMO; however, the processes producing these high concentrations of ferrous iron are unclear. Ferrous iron can be liberated from rocks via abiotic dissolution of iron oxides (11). Alternatively, our thermodynamic models suggest that microbial metabolisms which produce ferrous iron are favorable within biofilm communities on the surrounding rock surfaces. These microbial mechanisms include nitrate-dependent pyrite oxidation and ferric oxyhydroxide reduction with organic carbon, both of which are energetically favorable metabolisms at iron-rich DeMMO sites. Previous experiments with individual minerals indicate that pyrite and iron oxides at DeMMO can support biomass orders of magnitude greater than other minerals or inert surfaces, e.g., calcite or glass, and biofilms were found to selectively colonize ferrous iron-rich minerals in native rock (10, 12). Given these observations, bioleaching of the abundant pyrite and/or iron oxides in DeMMO host rock may be a widespread process at DeMMO that has the potential to contribute significantly to ferrous iron concentrations in fracture fluids.

Although unraveling the relative contributions from biotic or abiotic production of ferrous iron is difficult, this work reveals widespread metabolic potential for iron cycling across all DeMMO sites and across a diverse suite of taxa. Comparison of functional gene and taxonomic profiles between DeMMO and control communities indicates that DeMMO communities are not strongly influenced by *in situ* anthropogenic or surficial processes. Housekeeping genes represent a higher proportion of DeMMO communities than of the surface control community, perhaps due to competition for iron for fundamental cellular processes and as a major source of metabolic energy or to resistance to iron toxicity in response to high iron concentrations here. Specifically, high relative abundances of genes related to siderophore transport potential suggest the coping capacity for iron limitation through uptake of iron-scavenging siderophores (13, 14), and high relative abundances of genes related to iron regulation suggest the potential to cope with toxic iron concentrations (15, 16).

We observed trends in the abundance and distribution of genes for iron oxidation and reduction pathways that are consistent with local geochemistry and energy density estimates. Thermodynamic models suggest that organic carbon, nitrate, and oxygen should be important electron donors and acceptors for iron cycling at iron-rich sites D1 to D3 and D6. Genes specific to iron oxidation or reduction align with ferrous iron concentrations and redox potential, and genes for organic carbon oxidation, nitrate reduction, and oxygen reduction were detected at each of these sites. Iron cycling genes represent a large proportion of energy-related metabolic potential among DeMMO metagenomes, suggesting that metabolic reactions with iron are likely important strategies for energy conservation for planktonic members of the communities where iron concentrations are high.

### Iron-fueled life at DeMMO.

The potential for iron oxidation exists across all subsurface sites; however, the specific genes related to iron oxidation differ among sites. The shallower iron-rich sites which are relatively less reducing, D1 to D3, contain the highest relative abundances of genes for iron oxidation. Total iron concentrations are highest at D1, and fluids here are relatively oxidizing, with a redox potential of −61 mV. D1 iron oxidation genes are dominantly from Cyc2 cluster 1, characteristic of neutrophilic iron oxidizers (17). Indeed, two MAGs from D1 are assigned to the family *Gallionellaceae*, known for the ability to carry out autotrophic, microaerophilic iron oxidation in freshwater systems (18). Although present in low relative abundance, genes corresponding to Cyc2 cluster 1 were detected in MAGs from D2, D4, and D5 assigned to members of the family *Rhodocyclaceae*, previously associated with microaerophilic iron oxidation in iron-rich groundwaters (19). Cyc2 genes among D2 to D6 and service water control communities are dominantly those corresponding to representative cluster 3 associated with the genus *Leptospirillum*, within which iron bioleaching members have been implicated (17, 20). Cyc2 is a common attribute among DeMMO communities, suggesting the widespread potential for iron oxidation in DeMMO communities.

Of the DeMMO sites, D2, D3, and D6 are most similar with respect to iron energy metabolism potential, sharing many of the same genes for iron reduction and oxidation in similar proportions among these genes. Specifically, genes for iron reduction, including those encoding OmcS and OmcZ, and DFE are most abundant at D2, D3, and D6 and were not detected at D1. However, these genes represent the greatest proportion of the full metagenome at D3, suggesting the importance of iron as an electron acceptor at this site. The DFE genes encode multiheme cytochromes identified in Desulfovibrio ferrophilus involved in extracellular electron transport (21). OmcS and OmcZ genes encode c-type cytochromes implicated in extracellular reduction of Fe(III) in Geobacter sulfurreducens biofilms (22, 23). The high relative abundances of these genes suggest planktonic communities at D2, D3, and D6 are poised for biofilm formation and metabolic access to mineral substrates. Members within the class *Thermodesulfovibrionia* and family *Desulfobulbaceae* are associated with iron reduction across these sites and both possess DFE, OmcS, and OmcZ genes, supporting previous findings that these are important biofilm-forming taxa thought to be carrying out metal reduction on iron- and manganese-bearing minerals at DeMMO (10).

D4 and D5 are distinct from each other and from other sites in terms of the diversity of genes for iron cycling and their relative proportions. Among sites with iron reduction capacity, genes for energy metabolism with iron represent the lowest proportion of the full metagenome, suggesting that iron is not as important an energy source here. Further, these genes are represented by only a few hypothetical proteins for iron reduction at D4 and D5 and DFE at D5. This is supported by energy density estimates indicating that reactions with ferrihydrite or aqueous ferrous iron are much less favorable in D4 and D5 fluids relative to the other DeMMO sites. We did not detect measurable iron concentrations in D4 or D5 fluids; however, these sites have ∼10 to 60 times more sulfide than other DeMMO sites (2). Sulfide reacts quickly and spontaneously with ferrous iron to produce insoluble ferrous sulfide, removing aqueous iron from solution (24). Thus, any aqueous iron that may be present at D4 and D5 is likely removed very quickly by abiotic processes and is not a viable source of energy for the microbial communities here.

Several taxa appear to be generalists capable of both iron oxidation and reduction at DeMMO, including members of the candidate phylum “*Candidatus* Zixibacteria” and the order *Ignavibacteriales*. Members of “*Candidatus* Zixibacteria” were also previously found to be dominant members of both biofilm and planktonic DeMMO communities (9, 10, 12). While members of “*Candidatus* Zixibacteria” have not been isolated in culture and thus their optimal growth conditions are unknown, their persistence across geochemical conditions suggests their metabolic flexibility and importance as potential iron cycling members in the continental subsurface. Members of the order *Ignavibacteriales* dominate putative iron cycling taxa at DeMMO, representing 25% of MAGs where iron oxidation or reduction genes were detected. Members of the *Ignavibacteriales* are known obligate heterotrophs and were enriched in D6 biofilms (10, 25). Taken together, our data suggest that iron is a potentially important source of energy for heterotrophic, autotrophic, planktonic, and biofilm-forming members of DeMMO communities. Given its ubiquity, iron likely plays an important role for habitability across the deep continental biosphere, where iron is utilized as an energy source across a wide diversity of taxonomic groups beyond those classically associated with iron cycling, including members of the candidate phyla.

### FeGenie detects previously missed iron cycling genes.

Previous metagenomic surveys at DeMMO found no indication of iron cycling at sites D6 and DuselD located at a depth of 4,850 feet (8). This was unexpected given iron concentrations as high as 7 mg/liter Fe^2+^ at DuselD, high relative abundances of putative iron cycling taxa detected via 16S rRNA gene amplicon sequencing, and high energy yield estimated for ferrous iron oxidation (7). Our reannotation of the data from reference 8 reveals iron cycling potential on a par with that of the D6 community in this study (Fig. 2 and 5). There are several possible explanations for the lack of detected iron cycling genes in the previous study (8), including potential differences in sample preparation, sequencing or assembly methods, or annotation methods.

Differences in sample preparation are not likely to be a major contributing factor given that the only differences between our approach and that of the previous study (8) include filter mesh size (0.2 μm in the previous study versus 0.1 μm in this study) and volume of fluids sampled (8 liters in the previous study versus 10 liters in this study). Smaller filter meshes have been shown to capture ultrasmall cells characteristic of the candidate phylum radiation and the DPANN superphylum (26, 27). However, many of the taxa implicated in iron cycling in communities from this study are not associated with these groups, e.g., members of the *Thermodesulfovibrionia*, *Gallionellaceae*, *Rhodocyclaceae*, and “*Candidatus* Zixibacteria,” and are present in 16S rRNA gene amplicon surveys from fracture fluids collected on 0.2-μm filters (9, 10). Thus, while the larger meshes likely captured less diversity in the previous study (8), it does not seem likely that the filter size excluded biomass from all iron-cycling taxa. Otherwise, the equipment used for sample collection and DNA extraction methods and the final DNA yields are consistent for both sampling campaigns, and slight differences in total biomass sampled are not likely to result in a bias against recovery of iron-cycling taxa.

Both data sets were sequenced on an Illumina HiSeq platform; however, differences exist between the assembly methods, e.g., reads from data in the previous study (8) were assembled via IDBA-UD v1.1.1, whereas reads from this study were assembled with MEGAHIT. Taxon biases are certainly an important caveat to consider when comparing metagenomic assemblers; however, these biases are typically a function of high variance in taxon abundances (28, 29). DeMMO communities are temporally stable (9), and thus, it is not expected that community structures would be significantly different between sampling dates such that it would propagate taxon biases and result in the exclusion of all iron-cycling taxa from the previous assembly (8).

The most likely explanation for the lack of detected iron cycling genes is the annotation pipeline used. The genomes from the previous study (8) were annotated via the JGI IMG-MER pipeline in 2015. The JGI databases are updated constantly, so it should be expected that a data set annotated in 2015 would yield different results than the same data set annotated years later. However, our data set was annotated with JGI IMG-MER in 2019, and only two “OmcA/MtrC family decaheme *c*-type cytochrome” genes were detected among D6 MAGs (30). Further, we annotated this data set with METABOLIC and found only 5 hits (0.001% total metagenome) for “Metal reduction” associated with MtrB/MtrC genes within the full D6 metagenome. In contrast, when we used FeGenie to annotate both data sets, we detected a similar suite of iron cycling genes at D6 at both time points where genes for energy-related iron cycling make up as much as 0.045% of a given metagenome and are present in as many as 25% of MAGs binned from the metagenomic assemblies (Fig. 1; Fig. S4). MAGs from the previous study (8) were binned from a coassembly of D6 and DuselD sequence data; therefore, we cannot attribute individual taxa to a specific locality. However, the taxa associated with iron cycling in the coassembly are consistent with those from our study, including members of the “*Candidatus* Zixibacteria,” *Desulfobacteraceae*, “*Candidatus* Omnitrophica,” and *Ignavibacteriales* that are broadly capable of both iron oxidation and reduction (Fig. S4). Compared with other energy metabolisms, we find that not only is iron likely an important source of energy for DeMMO communities, but also, the potential for energy metabolisms with iron is on par with that for sulfur, previously found to be a major source of energy for the microbial communities here (8) (Fig. S2). These results suggest that the JGI IMG-MER and METABOLIC pipelines are not optimal for detecting iron cycling genes. In contrast, the FeGenie profile HMM database is highly curated and optimized for detection of iron cycling genes and has proved to be the best tool for this task in the case of DeMMO metagenomes. Given these findings, we recommend a combination of annotation pipelines for functional gene surveys where the detection of iron cycling genes is a major goal.

### Conclusion.

We probed the potential for iron-fueled life in a deep continental setting: the Deep Mine Microbial Observatory. We found that iron is an important source of energy at DeMMO for both the biofilms and the planktonic microbes in an otherwise energy limited system. While iron concentrations are locally high, competition for iron is likely also high due to its use in both critical cellular functions as well as energy metabolisms. Several key taxa possess flexible iron energy metabolisms and lifestyles, e.g., planktonic and attached, that likely aid their survival here. Where previous metagenomic surveys indicated no iron cycling potential at DeMMO, our study reveals that not only is iron cycling possible, energy metabolisms with iron represent a large proportion of functional genes among DeMMO metagenomes. Based on our findings, we recommend the use of multiple annotation pipelines or pipelines that are optimized for iron-related gene detection where characterizing iron-fueled life is a major goal. Given the ubiquity of iron in deep continental settings, iron-fueled life likely pervades the deep continental biosphere.

## MATERIALS AND METHODS

### Site description.

DeMMO is located in the Sanford Underground Research Facility (SURF), formerly the Homestake gold mine, in Lead, SD, USA. Depth and geochemical data for each of the six DeMMO sites are reported in Table 1 (2). Fracture fluids at sites D1, D3, and D6 are particularly iron rich, in contrast to sulfide-rich sites D4 and D5. Our surface control site is Whitewood Creek (WC), a surficial creek located proximal to SURF that drains into the aquifer system encompassing DeMMO (sampled collected at 44.364721 N, 103.733112 W) (31). Our *in situ* anthropogenic control is mine service water used for maintenance at SURF, e.g., for drilling operations, referred to here as service water (SW). A detailed description of DeMMO and geochemical data collection can be found in reference 2.

**TABLE 1:** Fracture fluid chemistry from April 2018

Parameter	Value for site					
	D1	D2	D3	D4	D5	D6
Depth (m)	244	244	610	1250	1478	1478
Temp (°C)	10.5	12	16.1	22.2	31.6	20.1
pH	7.3	7.7	7.1	8.3	8.6	8.2
ORP (mV)^*a*^	−68	−100	−61	−200	−149	−198
Concn (mg/liter) of:						
DOC	0.468	0.393	0.25	0.244	0.316	0.286
NO_3_^−^	0.3	0.3	0.3	1.1	0.7	0.3
NH_4_^+^	0.06	0.03	0.21	1.36	0.48	0
Fe^2+^	2.32	0.31	2.86	0	0	1.47
Total Fe	6.08	0.35	3.30	0.03	0.00	2.44
S^2−^	0.006	0	0.005	0.582	0.254	0.013
DO	0.025	0.054	0.029	0.031	0.015	0
SO_4_^2−^	393	84.8	1800	315	186	4110
Cl^−^	15.8	18.5	17	23.7	21.3	213
CH_4_	7.75E−06	5.85E−06	6.88E−05	7.19E−04	3.46E−04	5.04E−03
H_2_	1.19E−07	1.42E−07	2.68E−07	2.16E−07	1.98E−07	3.05E−07
CO_2_	0.023	0.018	0.092	0.013	0.005	0.002
CO	5.56E−06	4.94E−06	8.19E−06	3.54E−06	4.14E−06	2.52E−06

a ORP, oxidation-reduction potential.

### Metagenome analysis and annotation.

Fracture fluids were previously sampled in April 2018, and DNA extraction, sequencing, and metagenome assembly are described in detail elsewhere (30). In brief, we filtered 10 liters of fluids from DeMMO and control sites on 47-mm, 0.1-μm Supor filters. Filters were immediately frozen on dry ice in the field. DNA was extracted from the filters using a phenol-chloroform method and sequenced on an Illumina HiSeq2500 platform at Argonne National Laboratory. Paired-end reads were joined and trimmed with Trimmomatic 0.36 with default parameters and a minimum sequence length of 36 bp (30). Reads were assembled with a 1,000-bp minimum contig length using MEGAHIT (32). Metagenome-assembled genomes (MAGs) were generated using MetaBAT2 (33). MAG completeness and contamination were calculated using CheckM (34). Taxonomies were assigned to MAGs using GTDB-tk (35).

The metagenomic assemblies and MAGs were annotated with FeGenie v2.0 and METABOLIC v4.0 using amino acid files generated by Prodigal in the METABOLIC pipeline (6, 36). Contigs with annotations in both FeGenie and METABOLIC that are not assigned to both “Metal Reduction” in METABOLIC and “iron reduction” in FeGenie were reannotated as “ambiguous.” Ambiguous annotations were subtracted from the data set prior to analyses, totaling 49 ambiguous hits (0.13% of total hits annotated via METABOLIC and FeGenie, 0.002% of the total genes). Gene abundances were normalized to relative abundances by dividing the number of gene hits by the number of total genes in the metagenome for a given site. We categorize genes for iron oxidation and reduction as energy metabolism genes and genes for iron acquisition, storage, and regulation critical to cellular function as housekeeping genes (13, 37, 38). We performed hierarchical clustering on iron genes corresponding to iron oxidation or reduction annotated via FeGenie using between-group average linkage (unweighted pair group method using average linkages [UPGMA]) on Bray-Curtis dissimilarity indices visualized as a heat map using the vegan v 2.5-5, stats v 3.6.1, and gplot v 3.0.1.1 packages in R (39–41). METABOLIC annotations were recategorized into broader metabolic functions for the purpose of iron metabolism pathway interpretation (Table S1). A summary of metagenomic assembly and MAG statistics is available in Fig. S1.

In addition to annotating metagenome data from April 2018, we reannotated metagenome data from reference 8 with FeGenie. Fracture fluids were sampled in October 2013 from two borehole sites, D6 (formerly DuselB) and DuselD, each located at a depth of 1.5 km. Eight liters of fracture fluids was filtered on 47-mm, 0.2-μm Supor filters and immediately frozen on dry ice in the field. DNA was extracted from the filters and sequenced following the same protocols as the April 2018 field campaign. Sequences from D6 and DuselD were assembled separately and represent whole metagenomes, and MAGs were binned from a coassembly of sequences from both D6 and DuselB. Detailed assembly methods are described in reference 8, and corresponding fracture fluid chemistry sampling methods and data are described in reference 7. A summary of both April 2018 and October 2013 fluid chemistry is available in Table S2.

### Thermodynamic modeling.

We modeled the Gibbs free energy yield of 48 reactions with iron under *in situ* conditions at DeMMO in April 2018 as described in reference 10 (Table 2). In brief, using fracture fluid chemistry described in Table 1 (2), we calculated species activities from geochemical data using SPECE8 in Geochemist’s Workbench (42). Aqueous gas concentrations, K^+^ and NO_2_^−^ ion concentrations, and pH were averaged from measurements collected between December 2015 and December 2019 due to missing data for the April 2018 sampling trip. Perchlorate activities were estimated at equilibrium with Cl^−^. Acetate activities were estimated to be nearly equal to measured dissolved organic carbon (DOC) concentrations based on our preliminary data and reference 43. For aqueous CO, we substituted activities for concentrations averaged over six measurements taken between September 2016 and November 2017. We used CHNOSZ to calculate activity and equilibrium constants for each metabolic reaction (44). Gibbs free energy yields for each reaction were calculated as energy densities as in reference 7.

**TABLE 2:** Metabolic reactions with iron

Type and no.	Reaction^*a*^	No. of e^–^/reaction
Fe^2+^ as electron donor		
1	8Fe^2^ + ClO_4_^−^ + 12H_2_O ↔ 8FeOOH + Cl^−^ + 16H^+^	8
2	6Fe^2^ + CO + 11H_2_O ↔ 6FeOOH + CH_4_ + 12H^+^	6
3	8Fe^2^ + HCO_3_^−^ + 13H2O ↔ 8FeOOH + CH_4_ + 15H^+^	8
4	8Fe^2^ + 2HCO_3_^−^ + 12H2O ↔ 8FeOOH + acetate + 15H^+^	8
5	8Fe^2^ + NO_3_^−^ + 13H_2_O ↔ 8FeOOH + NH_4_^+^ + 14H^+^	8
6	8Fe^2^ + NO_3_^−^ + 13H_2_O ↔ 8FeOOH + NO_2_^−^ + 14H^+^	2
7	4Fe^2+^ + O_2_ + 6H_2_O ↔ 4FeOOH + 8H^+^	4
FeOOH as electron acceptor^*b*^		
8–10	8FeOOH + acetate + 15H^+^ ↔ 8Fe^2+^ + 2HCO_3_^−^ + 12H_2_O	8
11–13	8FeOOH + CH_4_ + 15H^+^ ↔ 8Fe^2+^ + HCO_3_^−^ + 13H_2_O	8
14–16	6FeOOH + CH_4_ + 12H^+^ ↔ 6Fe^2+^ + CO + 11H_2_O	6
17–19	2FeOOH + CO + 3H^+^ ↔ 2Fe^2+^ + HCO_3_^−^ + 2H_2_O	2
20–22	2FeOOH + H_2_ + 4H^+^ ↔ 2Fe^2+^ + 4H_2_O	2
23–25	2FeOOH + HS^−^ + 5H^+^ ↔ 2Fe^2+^ + S^0^ + 4H_2_O	2
26–28	6FeOOH + NH_4_^+^ + 10H^+^ ↔ 6Fe^2+^ + NO_2_^−^ + 10H_2_O	6
Hematite as electron acceptor		
29	4Fe_2_O_3_ + acetate + 15H^+^ ↔ 8Fe^2+^ + 2HCO_3_^−^ + 8H_2_O	8
30	4Fe_2_O_3_ + CH_4_ +15H^+^ ↔ 8Fe^2+^ + HCO_3_^−^ + 9H_2_O	8
31	3Fe_2_O_3_ + CH_4_ +12H^+^ ↔ 6Fe^2+^ + CO + 8H_2_O	6
32	Fe_2_O_3_ + CO +3H^+^ ↔ 2Fe^2+^ + HCO_3_^−^ + H_2_O	2
33	Fe_2_O_3_ + H_2_ +4H^+^ ↔ 2Fe^2+^ + 3H_2_O	2
34	Fe_2_O_3_ + HS^−^ +5H^+^ ↔ 2Fe^2+^ + S^0^ + 3H_2_O	2
35	3Fe_2_O_3_ + NH_4_^+^ +10H^+^ ↔ 6Fe^2+^ + NO_2_^−^ + 7H_2_O	6
Magnetite as electron acceptor		
36	4Fe_3_O_4_ + acetate + 23H^+^ ↔ 12Fe^2+^ + 2HCO_3_^−^ + 12H_2_O	8
37	4Fe_3_O_4_ + CH_4_ + 23H^+^ ↔ 12Fe^2+^ + HCO_3_^−^ + 13H_2_O	8
38	3Fe_3_O_4_ + CH_4_ +18H^+^ ↔ 9Fe^2+^ + CO + 11H_2_O	6
39	Fe_3_O_4_+ CO + 5H^+^ ↔ 3Fe^2+^ + HCO_3_^−^ + 2H_2_O	2
40	Fe_3_O_4_+ H_2_ + 6H^+^ ↔ 3Fe^2+^ + 4H_2_O	2
41	Fe_3_O_4_+ HS^−^ +7H^+^ ↔ 3Fe^2+^ + S^0^ + 4H_2_O	2
42	3Fe_3_O_4_ + NH_4_^+^ +16H^+^ ↔ 9Fe^2+^ + NO_2_^−^ + 10H_2_O	6
Pyrite as electron donor		
43	3FeS_2_ + 8CO + 16H_2_O ↔ 3Fe^2+^ + 6SO_4_^2−^ + 8CH_4_	48
44	3FeS_2_ + 2HCO_3_^−^ + 2H_2_O + 2H^+^ ↔ Fe^2+^ + 2SO_4_^2−^ + 2CH_4_	16
45	FeS_2_ + 8NO_3_^−^ ↔ Fe^2+^ + 2SO_4_^2−^ + 8NO_2_^−^	16
Siderite as electron donor		
46	6FeCO_3_ + CO + 11H_2_O ↔ 6FeOOH_fer_ + 6HCO_3_^−^ + CH_4_ + 6H^+^	6
47	2FeCO_3_ + NO_3_^−^ + 3H_2_O ↔ 2FeOOH_fer_ + NO_2_^−^ + 2HCO_3_^−^ + 2H^+^	2
48	8FeCO_3_ + SO_4_^2−^ + 12H_2_O ↔ 8FeOOH_fer_ + HS^−^ + 8HCO_3_ + 7H^+^	8

a Aqueous forms were used for H_2_, CH_4_, and CO. DOC concentrations were used to estimate acetate (C_2_H_3_O_2_^−^).

b FeOOH modeled as ferrihydrite, goethite, and lepidocrocite corresponding to each of the three reaction numbers per reaction, respectively.

### Data availability.

All code and corresponding data for this study are available at github.com/CaitlinCasar/Casar2021_DeMMO_IronFueledLife. Sequence data for April 2018 and October 2013 metagenomic assemblies and their respective MAGs were submitted to NCBI (BioProject number PRJNA563685) and JGI-IMG (no. 3300007351) (8, 30), and their metadata can be found in reference 30.
