# Supplementary material for: Iron-Fueled Life in the Continental Subsurface: Deep Mine Microbial Observatory, South Dakota, USA
Source: Appl Environ Microbiol. 2021 Sep 28;87(20):e00832-21. doi: 10.1128/AEM.00832-21 (PMC8478452; doi:10.1128/AEM.00832-21)
Supplement: Supplemental file 1 — Figures S1 to S4, supplemental table legends. Download AEM.00832-21-s0001.pdf, PDF file, 2.7 MB [file aem.00832-21-s0001.pdf]

Supplementary Figures

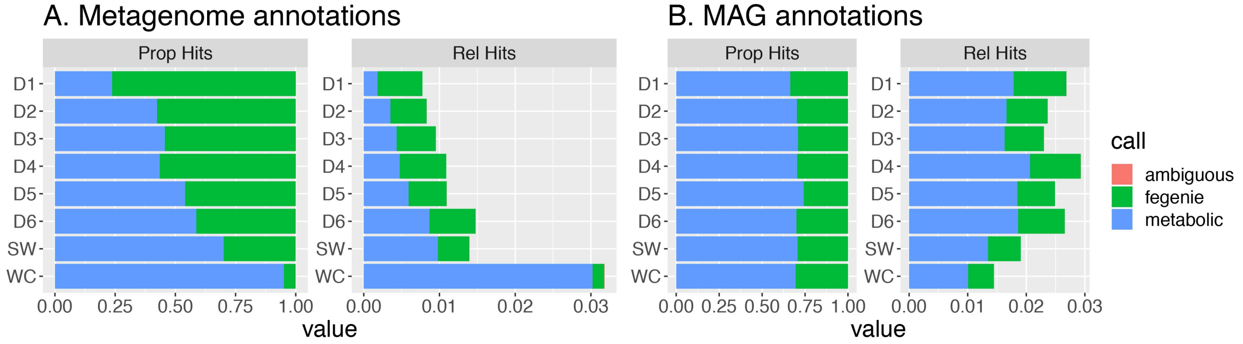

C. Metagenome Stats

	Gene Counts
D1	128801
D2	378180
D3	338355
D4	178788
D5	190877
D6	444350
SW	565484
WC	543229

D. MAG Stats

	80% Complete	Complete Fe Cyclers	Fe Cyclers	Total Genes	Total Genomes
D1	3	1	3	47550	24
D2	16	5	23	149567	96
D3	23	10	15	152201	97
D4	8	2	4	74785	44
D5	9	3	7	108447	56
D6	36	10	16	221510	97
SW	30	3	7	204649	93
WC	2	0	0	68658	57

Supplementary Figure 1. Proportions of functional gene annotations from FeGenie, METABOLIC, or both (ambiguous). **A-B)** Proportion of functional gene annotations among only functional genes (left) and among all genes (right) for metagenomic assemblies and metagenome assembled genomes, respectively. **C-D)** Summary statistics for metagenomic assemblies and metagenome assembled genomes, respectively.

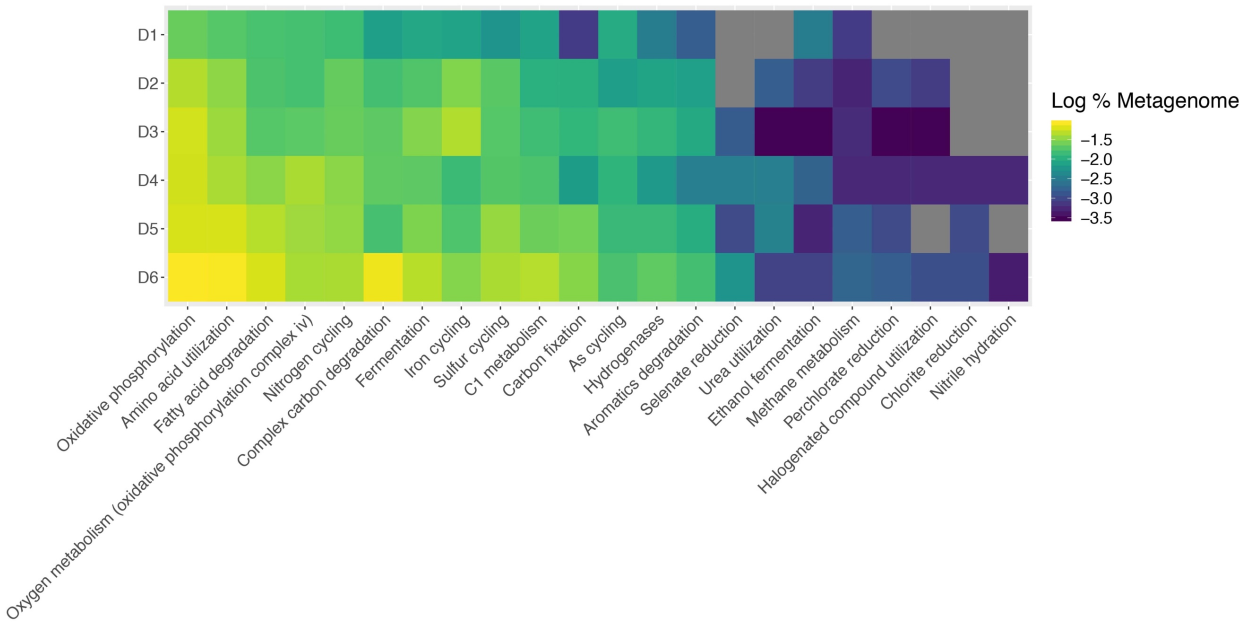

Supplementary Figure 2. Heatmap of DeMMO community energy metabolism pathways annotated via FeGenie and METABOLIC. Scale is log of relative abundances of genes per metagenome, grey indicates that the pathway was not

detected via FeGenie or METABOLIC pipelines. ‘Iron Cycling’ includes ‘iron oxidation’ and ‘iron reduction’ categories from FeGenie and ‘Metal reduction’ from METABOLIC.

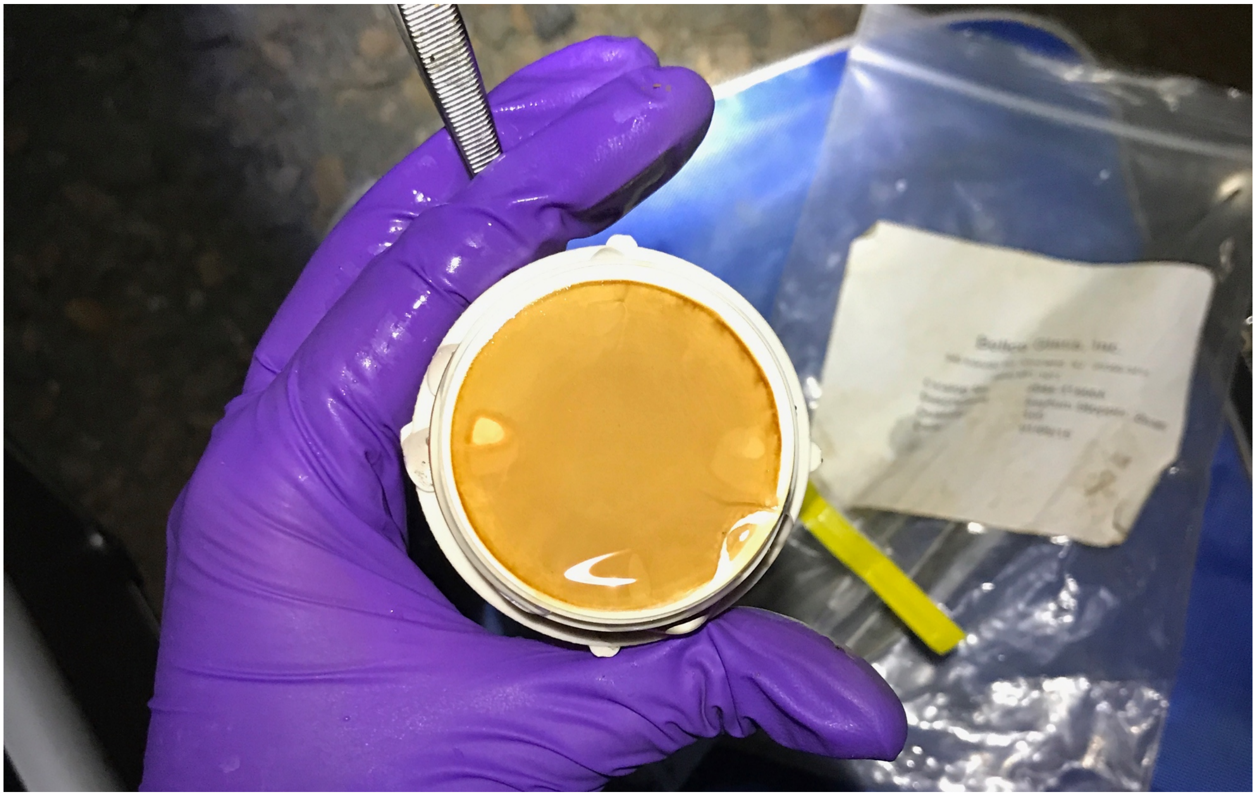

Supplementary Figure 3. Visible iron floc on a Supor filter after filtering iron-rich fracture fluids at DeMMO.

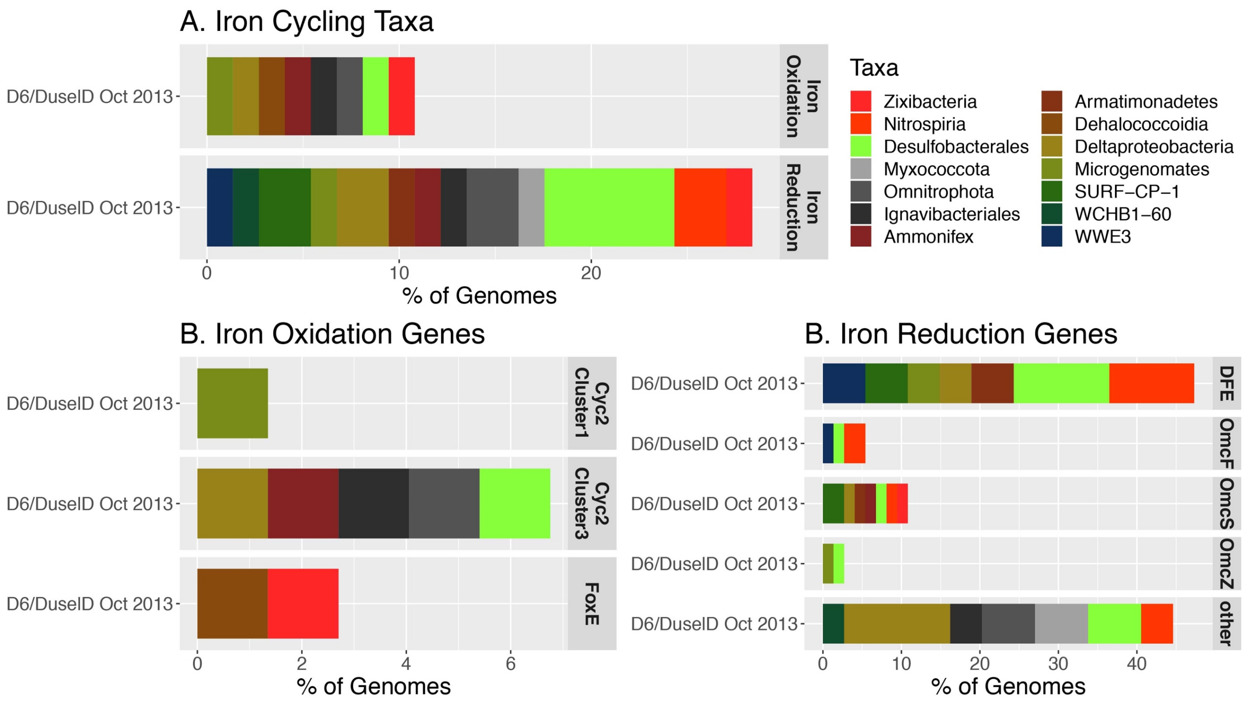

Supplementary Figure 4. Relative abundances of iron cycling taxa from October 2013 sampling trip annotated via FeGenie. MAGs were binned from a co-assembly of D6 and DuselD metagenomes. Only FeGenie categories "iron oxidation" and "iron reduction" are shown. "Other" gene category represents hypothetical proteins attributed to porins and cytochromes for iron reduction.

Supplementary Tables

Supplementary Table 1. METABOLIC annotations recategorized into broader metabolic functions for the purpose of iron metabolism pathway interpretation.

Supplementary Table 2. DeMMO site geochemistry from April 2018 and October 2013 sampling campaigns.

Supplementary Table 3. DeMMO whole metagenome assemblies annotated with FeGenie and METABOLIC from April 2018 and October 2013 sampling campaigns.

Supplementary Table 4. Abbreviations corresponding to iron oxidation and reduction genes annotated via FeGenie referenced in Figure **Figure 2**.

Supplementary Table 5. DeMMO metagenome-assembled genome metadata from April 2018 sampling campaign.

Supplementary Table 6. DeMMO thermodynamic modeling results for metabolisms with iron.
